# Effects of the Ethyl Acetate Fraction of *Alchornea triplinervia* on Healing Gastric Ulcer in Rats

**DOI:** 10.3390/ph4111423

**Published:** 2011-10-25

**Authors:** Zeila P. Lima, Flavia Bonamin, Tamara R. Calvo, Wagner Vilegas, Lourdes C. Santos, Ariane L. Rozza, Claudia H. Pellizzon, Lucia R. M. Rocha, Clélia A. Hiruma-Lima

**Affiliations:** 1 Department of Physiology, Univ. Estadual Paulista-UNESP, Rubião Junior, cp 510, CEP 18618-000, Botucatu, São Paulo, Brazil; 2 Institute of Chemistry, Univ. Estadual Paulista-UNESP, cp 355, CEP 14801-970, Araraquara, São Paulo, Brazil; 3 Department of Morphology, Univ. Estadual Paulista-UNESP, cp 610, CEP 18618-000, UNESP, Botucatu, São Paulo, Brazil

**Keywords:** *Alchornea triplinervia*, Euphorbiaceae, healing ulcer effect, phenolic compounds, angiogenesis

## Abstract

*Alchornea triplinervia* (Spreng.) Muell. Arg (Euphorbiaceae) is a medicinal plant commonly used by people living in the Cerrado region of Brazil to treat gastrointestinal ulcers. We previously described the gastroprotective action of methanolic extract (ME) of *Alchornea triplinervia* and the ethyl acetate fraction (EAF) in increasing of prostaglandin E_2_ (PGE_2_) gastric levels in the mucosa. In this work we evaluated the effect of EAF in promoting the healing process in rats with acetic acid-induced gastric ulcers. In addition, toxicity was investigated during treatment with EAF. After 14 days of treatment with EAF, the potent stimulator of gastric cell proliferation contributed to the acceleration of gastric ulcer healing. Upon immunohistochemical analysis, we observed a pronounced expression of COX-2, mainly in the submucosal layer. The 14-day EAF treatment also significantly increased the number of neutrophils in the gastric mucosa regeneration area. The EAF induced angiogenesis on gastric mucosa, observed as an increase of the number of blood vessels supplying the stomach in rats treated with EAF. Oral administration for 14 days of the ethyl acetate fraction from *Alchornea triplinervia* accelerated the healing of gastric ulcers in rats by promoting epithelial cell proliferation, increasing the number of neutrophils and stimulation of mucus production. This fraction, which contained mainly phenolic compounds, contributed to gastric mucosa healing.

## 1. Introduction

Gastric ulcers are a serious problem in many parts of the World. The aetiology of gastroduodenal ulcers is influenced by various factors and ulcers are worsened by inadequate dietary habits, excessive ingestion of non-steroidal anti-inflammatory drugs (NSAIDs), stress, hereditary predisposition and infection by *Helicobacter pylori* [1]. Several pharmaceutical products have been employed for the treatment of gastroduodenal ulcers and peptic diseases, resulting in decreased mortality and morbidity rates. However they are not completely effective and they produce many adverse effects [2]. Epidemiological studies suggest that a history of gastric ulcers is a risk factor for gastric cancer [3]. Hansson *et al*. [4] observed that a history of gastric ulcers increases the cancer risk twofold. Despite the progress in conventional chemistry and pharmacology that has led to the production of more effective drugs, the plant kingdom might still represent a useful source of new anti-ulcer compounds for development as pharmaceutical entities or, alternatively, as adjuncts to existing therapies [5,6].

When the selection of plants for therapies is made on the grounds of their traditional use, the chances of success are greater [7]. An ethnopharmacological inventory taken in the Cerrado formation of central Brazil revealed a number of medicinal plants used to treat gastric pain and gastritis. According to this inventory the leaves and other aerial parts of *Alchornea triplinervia* are commonly used in folk medicine as a tea to treat gastric disturbances [8]. Recently, we reported the antisecretory, anti-*Helicobacter pylori* and gastroprotective effects of this species [9]. The ethyl acetate fraction (EAF) obtained from this extract at doses of 100 mg/kg also showed gastroprotective effects by increasing PGE_2_ levels in rodent gastric mucosa [9].

The present work was carried out to investigate the healing property of the EAF obtained from *Alchornea triplinervia* leaves by oral administration for 14 consecutive days to rats with acetic acid-induced ulcers. The healing effect of the EAF was evaluated by morphometric and immunohistochemical techniques and compared with the healing action induced by cimetidine (positive control) at the same dose. We also investigated the sub-acute toxicity of EAF and cimetidine in rats after oral administration for 14 days.

## 2. Experimental

### 2.1. Plant Material and Preparation of Ethyl Acetate Fraction

Leaves of *Alchornea triplinervia* (Spreng.) Müll. Arg were collected at Botucatu (São Paulo State, Brazil) in August 2003 and the vegetal species was identified by Jorge Tamashiro from the Institute of Biology at the University of Campinas, São Paulo State, Brazil. A voucher specimen (BOTU: 14873) was deposited at the herbarium of the Universidade Estadual Paulista, Campus Botucatu, Brazil. The leaves (500 g) of *Alchornea triplinervia* were air dried (7 days at 40 °C) and powdered. The powdered aerial parts were exhaustively extracted with methanol (1 L) successively at room temperature (three times, 72 h) to afford after removal of the solvent 75 g of extract (15%). A portion (28 g) of the methanol extract was partitioned between EtOAc/H_2_O (1 L, 1:1, v/v) yielding 7.5 g (27%) of ethyl acetate fraction (EAF).

### 2.2. Analytical and Quantitative Measurement of Total Phenolic Compounds by HPLC-UV-PDA

In the aqueous and ethyl acetate fractions, the flavonoid concentration was determined as follows: an aliquot of each fraction (30 mg) was filtered in Sep-Pak cartridge (Sigma, C_18_) and analysed using a Varian ProStar HPLC system equipped with an RP-18 column (250 × 4.60 mm i.d., 5 μm, Phenomenex Luna). The mobile phase was a linear gradient of water (A) and acetonitrile (B), both with 0.05% trifluoroacetic acid, varying from 30 to 70% of B over 60 min at a flow-rate of 1.0 mL/min. The effluent was monitored using a ProStar 330 photodiode-array ultraviolet detection (UV-PDA) system at 360 nm. A stock solution (1 mg/mL) of rutin was prepared in methanol. The calibration curve was generated with seven different concentrations of rutin (10, 20, 50, 100, 200, 300 and 500 μg/mL) each measured in duplicate. To determine the total concentration of flavonoids, the peak areas were transformed to concentrations using the rutin calibration curve which was linear over the range of 10 to 500 μg/mL with a correlation coefficient of 0.9999. All data presented are mean ± standard deviation of four independent experiments (*n* = 4).

### 2.3. Animals

Male Wistar albino rats (150 to 250 g) from the central animal house of the UNESP were used. The rats were fed a certified Nuvilab^®^ (Nuvital) diet with free access to tap water under standard conditions (12 h dark-12 h light, 60 ± 1.0% humidity and 21 ± 1 °C). Prior to assays, the rats were fasted as drugs were administered orally (by gavage) using 10 mL/kg of 8% Tween 80^®^ as the vehicle. Moreover, the rats were kept in cages with raised floors of wide mesh to prevent coprophagy. All experiments were performed in the morning, and following the recommendations of the Canadian Council on Animal Care [10]. The UNESP Institutional Animal Care and Use Committee approved all of the employed protocols.

### 2.4. Healing of Acetic Acid-Induced Gastric Lesions

The experiments were performed according to the method described by Takagi *et al.* [11] with some modifications as suggested by Okabe and Amagase [12]. Three groups of male Wistar rats fasted for 24 h were used in this experiment (n = 4 to 5 per group). Under anaesthesia, a laparotomy was done in all animals through a midline epigastric incision. After exposing the stomach, 0.05 mL (v/v) of a 30% acetic acid solution was injected into the subserosal layer in the glandular part of the anterior wall. The stomach was bathed with saline (20 °C) to avoid adherence to the external surface of the ulcerated region. The abdomen was then closed and all the animals were fed normally. We selected the lower effective dose (100 mg/kg) of the ethyl acetate fraction (EAF) of *Alchornea triplinervia* that exhibited gastroprotective action in experimental assays performed by Lima *et al.* [9]. Another two groups received cimetidine (100 mg/kg, positive control group) or vehicle (10 mL/kg, negative control group). All treatments were administered orally once a day for 14 consecutive days beginning one day after surgery. Body weight was recorded daily throughout the experiments and the macroscopic analyses and weight of some organs (liver, kidney, heart, spleen and lungs) were compared among the different treatments to evaluate the possible subchronic toxicity induced by them. On the day after the last drug administration, the rats were euthanized and the stomachs were removed. The gastric lesions were evaluated by examining the inner gastric surface with a dissecting magnifying glass. The macroscopic ulcer area was evaluated by examining the external and internal lesion area with digital caliper (Mitutoyo, Brazil) was subsequently determined as described by Takagi *et al.* [11].

Biochemical analysis: For the biochemistry analysis, the blood of rats submitted from different treatments was collected immediately after the dead by bleeding and blood samples were reserved in specific tubes (5 mL) and were submitted to centrifugation (2,000 × g for 10 min). After the centrifugation, the serum was stored at −20 °C until the biochemical analyses. An automated biochemical analyser (SBA-200, CELM, Brazil) was used to measure serum biochemical parameters such as urea, creatinine, aspartate aminotransferase (AST) alanine aminotransferase (ALT).

Histology methods: The stomachs of the rats subjected to the different treatments were removed and opened to expose the ulcer. The lesion was sectioned, and fixed in ALFAC solution (alcohol 900 mL, chloroform 100 mL and acetic acid 50 mL) for 24 h at 4 °C. Then the samples were processed for routine embedding in paraplast and cut into 7-μm section. The slides were observed after staining with haematoxylin and eosin (H&E) [13] and periodic acid-Schiff (PAS) [14] to observe mucus production. Then, neutrophils in the submucosa under the lesion were counted and angiogenesis was measured at 32,000× magnification (Leica microscope). The number of positively stained vessel cells (dark brown) at the ulcer margin was counted. The samples were analysed with a Leica microscope using the Leica Qwin Software (Leica-England).

Morphometric analyses: For the morphometric analyses, stomach slices were examined on the Leica microscope using the Leica Qwin Software, and measurements were made in normal and regenerating mucosal areas using a variation of method reported by Ishihara and Ito [15]. Immunohistochemistry: Representative sections were deparafinised, rehydrated and immunostained using the ABC method. Nonspecific labeling was blocked with H_2_O_2_ and goat serum prior to incubation with antisera specific for proliferating cell nuclear antigen (PCNA, Novacastra and COX-2 (Caymen Chemical). After rinsing in phosphate-buffered saline (PBS 0.01 mol/L, pH 7.4), the sections were incubated in secondary antiserum (ABC kit). After washing the slides with PBS the ABC complex was prepared and the revelation reaction was carried out in a 3,3′-diaminobenzidine-tetrahydrochloride (DAB solution) containing 0.01% H_2_O_2_ in PBS. After immunostaining, the sections were lightly counterstained with Mayer's hematoxylin and the immunoreactive cells were observed on a Leica microscope using Leica Qwin Software. As a control some slides were processed omitting the primary antibody and other slides omitting the primary and secondary antibodies. These procedures were done for PCNA (Novo Castra) and COX-2 (Cayman Chemical). Twenty sections were examined for each antibody. Immunolabelling of PCNA was measured with the aid of AVSoft BioView^®^ software.

### 2.5. Statistical Analysis

Results were expressed as means ± S.E.M. Statistical significance was tested by one-way analysis of variance followed by Dunnett's test with the significance threshold of *p* < 0.05.

## 3. Results and Discussion

**Figure 1.**
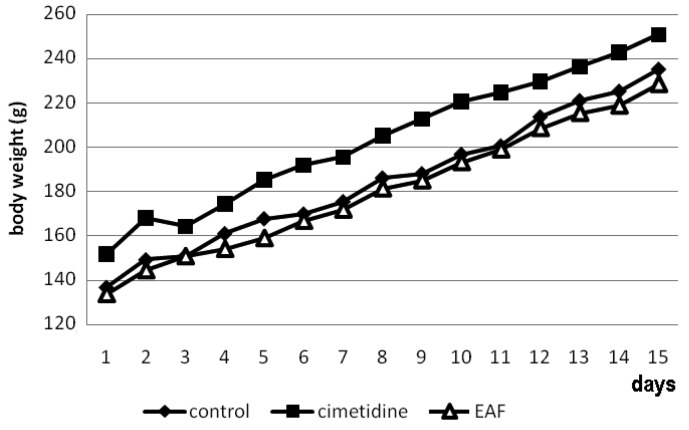
Effects on body weight of a single dose (100 mg/kg, p.o.)/day of ethyl acetate fraction of *Alchornea triplinervia* leaves (EAF) administered during 14 consecutive days after ulcer formation (n = 6). Results are expressed as mean of body weight (g).

The leaves and aerial parts of *Alchornea triplinervia* are commonly used in folk medicine as a tea to treat gastric disturbances and we have reported the antisecretory, anti-*Helicobacter pylori* and gastroprotective effects of this species [9]. The EAF obtained from methanolic extract contained almost 15 times more flavonoids by concentration than did the aqueous fraction, and acute administration of the EAF increased PGE_2_ levels in the gastric mucosa [9]. In this study we evaluated the ability of the EAF to heal ulcers during a 14 consecutive day-treament. The evaluation of ulcer healing using the model described by Takagi *et al*. [11] is a well established assay, and this experimental model highly resembles human ulcers in terms of both pathological features and healing mechanisms. Rats subjected to different treatments in this gastric ulcer model were also evaluated for some important toxicological parameters, such as change in body weight during the 14-day treament, mortality, some vital organ weight and biochemical parameters (Figure 1 and Table 1). Over the 14 days, body weight of the EAF group did not differ significantly from that of the vehicle group. In addition, the average weight of the vital organs and the visceral conditions were normal in the EAF group compared to those of the control group (*p* > 0.05).

That none of the treatments resulted in death suggests the absence of lethal effect which was corroborated the biochemical serum analyses showing no significant alterations in the parameters measured (Table 1). Together, these results suggest that the EAF might have utility as an anti-ulcer agent. Macroscopic analyses of acetic acid-induced gastric lesion did not reveal significant differences among the EAF, cimetidine and vehicle groups (Table 2).

However, in cimetidine-treated rats (the positive control group) and EAF group, there was an decrease in the external lesion area compared with that of the vehicle-treated group (35 and 44% respectively). The values from macroscopic and histological analyses of normal and regenerating mucosal areas showed that the 14-day EAF treatment increased cicatrisation in normal mucosa tissue. This result was compatible with the observed reduction of the regenerating area in the EAF group.

**Figure 2.**
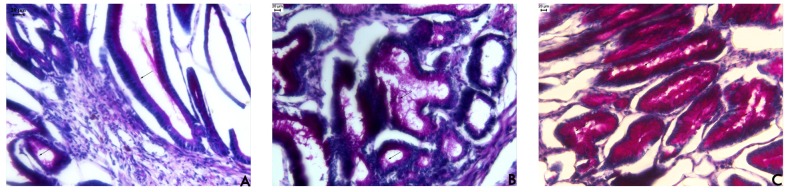
Photomicrography of histological analyses of stomach of rat treated orally with (**A**) vehicle (10 mL/kg); (**B**) cimetidine (100 mg/kg); and (**C**) acetate fraction of *Alchornea triplinervia* (100 mg/kg). The scale represents 20 μm and arrows denote mucus area by PAS method.

Hayashida *et al*. [16] observed a notable expression of mucin in regenerating areas in acetic acid-induced ulcers. The mucus bicarbonate barrier is the only pre-epithelial barrier between the lumen and epithelium, and when its breaks down in disease, other intracellular mechanisms come into play [17]. The periodic acid-Schiff (PAS) histochemical method exhibits characteristic carmine staining of stomach regions that secrete mucopolysaccharides. At the end of the treatment period, all stomachs were processed by PAS histochemical analyses. In samples from rats treated with EAF, we observed the intense secretion of mucus in gastric glands (Figure 2) that was absent in sample from the other groups. Mucus production represents one of the main mechanisms of local gastric mucosal defense [17]. A number of factors appear to influence ulcer healing, but mucus and bicarbonate secretion may be important in the ulcer healing process because the mucus/bicarbonate layer protects newly formed cells from acid and peptic injury [18].

Histologically, an ulcer consists of two major structures: a distinct ulcer margin formed by the adjacent non-necrotic mucosa (the epithelial component) and the granulation tissue at the base of the ulcer (the conjunctive tissue component). For ulcer healing, the action of many factors, including cell migration, cell proliferation, angiogenesis, all ultimately leading to scar formation, is necessary [19].

**Figure 3.**
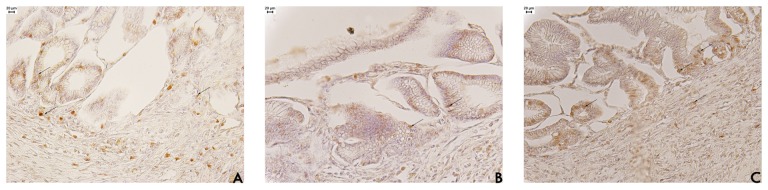
Photomicrography of histological analyses of stomach of rat treated orally with (**A**) vehicle (10 mL/kg); (**B**) cimetidine (100 mg/kg); and (**C**) acetate fraction of *Alchornea triplinervia* (100 mg/kg). The scale represents 20 μm and arrows denote nucleus PCNA positive by peroxidase method.

Cell proliferation plays a predominant role in wound healing, and PCNA (proliferation cell nuclear antigen) has been demonstrated to be a useful marker of cell proliferation. PCNA is an auxiliary protein of DNA polymerase, an enzyme necessary for DNA synthesis and cell proliferation. In the present study, factor related to tissue repair were examined in an attempt to gain information on the mechanism through which EAF affects ulcer healing. The results indicated that EAF stimulated gastric epithelial cell proliferation by enhanced expression of PCNA (Figure 3).

The positive PCNA immunohistochemical labeling in stomach tissue from EAF-treated rats appeared mainly at the base of the glands (Figure 3). This result occurs because stem cells re-organise at the base of glands, indicating the unidirectionality of cell proliferation, which is different in normal regions that have a bi-directional proliferation [20]. We also assessed the number of neutrophils in the region under the regenerative mucosa in stomach tissue from the three groups (Table 3). The cell counts showed a significant increase (*p* < 0.05) in neutrophils in regions under the regenerative mucosa for EAF (15.39 ± 1.52 cells/mm^2^) compared with the saline (6.40 ± 1.69 cells/mm^2^) or cimetidine (11.40 ± 0.93 cells/mm^2^) groups.

The increase in neutrophils observed in the regenerative mucosal region of rats treated with EAF might be an important local protective factor against bacterial agents or local infections. Such protection was mainly attributed to lysozymes from neutrophils that increased mucus production by PGE_2_ activation [21]. Lysozymes also stimulated the production of glutathione (GSH) in gastric mucosa cells which protects the cells from oxidative stress [22]. Another important process in ulcer healing is the formation of new vessels and microvessels at the ulcer base. This process is promoted in the granulation tissue within ulcer scar [23-25].

**Figure 4.**
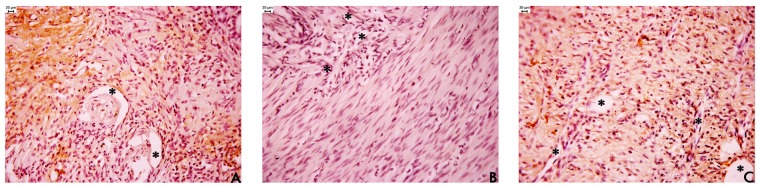
Photomicrographs submucosa from the stomach of rats subjected to different treatments. (**A**) vehicle (10 mL/kg); (**B**) cimetidine (100 mg/kg); and (**C**) ethyl acetate fraction of *Alchornea triplinervia* (100 mg/kg) immunostained for evidence of blood vessels and stained with hematoxylin and eosin for evidence the cellular structure. The asterisks (*) show the vessels lumen and scale represents 20 μm.

Our results (Table 3) show that EAF treatment increased the number of vessels in the ulcer margin compared to the other treatments, indicating that EAF stimulated angiogenesis in this regenerative area. Angiogenesis is essential for the healing of chronic gastroduodenal ulcers, and several new angiogenic factors have been identified in the gastric mucosa that play an organ-specific role in the formation of large blood vessels supplying the stomach and intestines [26,27]. This increased angiogenesis in the EAF group (Figure 4) explains why we observed increase in normal epithelial height and a reduction in regenerative epithelial height. The EAF treatment accelerated the cicatrisation process by increasing angiogenesis that contribute to an increase of the mucus barrier. This barrier plays an important role in the maintenance and renewal of the gastric mucosa epithelium.

To further elucidate the healing process, we measured COX-2 expression during ulcer healing. COX 2 is an important factor for epithelial cell proliferation, migration and re-epithelialisation and reconstruction of gastric glands. Gastric damage is the main side effect associated with inhibition of COX-2 [28]. Despite these clinical findings, there is evidence that COX-2 is protective within the gastrointestinal tract, perhaps explaining why in some studies use of COX-2-selective inhibitors can be associated with ulcer rates higher than placebo. Both COX-1 and COX-2 are expressed in human gastric mucosa, and COX-2-selective inhibitors suppress the formation of prostanoids from healthy samples of human gastric [29].

**Figure 5.**
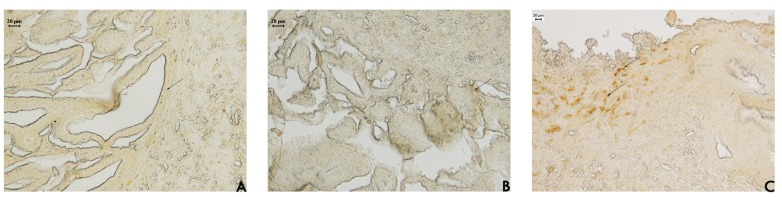
Photomicrographs submucosa from the stomach of rats subjected to different treatments. (**A**) vehicle (10 mL/kg); (**B**) cimetidine (100 mg/kg); and (**C**) ethyl acetate fraction of *Alchornea triplinervia* (100 mg/kg) immunostained of stomach of rat with COX-2 (scale represents 20 μm). The arrows denote cells COX-2 positive and note the high intensity of COX-2 label at lesion area in C.

In animal models of ulceration and gastrointestinal damage, COX-2 products actually promote gastrointestinal healing, and inhibition of COX-2 is required to produce acute gastrointestinal damage [30]. Guo *et al*. [31] showed that highly selective COX-2 inhibitors delayed ulcer healing impaired angiogenesis in rats. In this study, the expression of COX-2 was increased in gastric tissues obtained from rats in the EAF groups (Figure 5). A marked increase in COX-2 expression was found at the base of the ulcers. This result thus confirmed that COX-2 may play an essential role in the healing of gastric ulcers. Lima *et al*. [9] observed that EAF was able to increase PGE_2_ synthesis after acute treatment. Taken together, the action of EAF on COX-2 and PGE_2_ the gastric mucus barrier, cell proliferation and angiogenesis suggest that it might represent a new strategy of healing gastric ulcers.

## 4. Conclusions

In conclusion, oral administration of the ethyl acetate fraction (EAF) for 14 days accelerated the healing of gastric ulcer in rats by promoting epithelial cell proliferation, increasing neutrophil number and increasing mucus production. In immunohistochemical analyses we observed a large number of COX-2-expressing cells mainly in the submucosa and our results showed that EAF increases angiogenesis in healing gastric mucosa.

## Figures and Tables

**Table 1.** Effects on body and organ weights and serum biochemical parameters of a single dose (100 mg/kg, p.o.) of ethyl acetate fraction (EAF) obtained from leaves of *Alchornea triplinervia* administered for 14 consecutive days after ulcer formation.

**Treatment (p.o.)**		**Control**	**Cimetidine**	**EAF**
**Weight (g)**	**Body**	235.05 ± 9.65	250.82 ± 10.22	228.48 ± 10.90
	**Kidney**	1.69 ± 0.09	1.85 ± 0.06	1.72 ± 0.06
	**Lungs**	1.54 ± 0.10	1.47 ± 0.04	1.52 ± 0.12
	**Liver**	6.24 ± 0.86	6.86 ± 0.70	6.07 ± 0.75
	**Heart**	0.91 ± 0.05	1.00 ± 0.06	0.94 ± 0.04
	**Spleen**	0.65 ± 0.08	0.73 ± 0.08	0.64 ± 0.04

**Serum biochemical parameters**	**Urea (mg/dL)**	45.29 ± 1.75	41.83 ± 1.10	48.16 ± 4.03
	**Creatinine (mg/dL)**	0.88 ± 0.04	0.63 ± 0.04	0.72 ± 0.06
	**AST(UI/L)**	222.50 ± 11.23	244.57 ± 20.99	264.66 ± 18.03
	**ALT(UI/L)**	150.00 ± 8.60	112.82 ± 23.82	115.20 ± 3.90

Results are mean ± S.E.M. n = 6. ANOVA followed by Dunnett's test. No significance when *p* > 0.05.

**Table 2.** Effects of the 14-day treatment with ethyl acetate fraction (EAF) from *Alchornea triplinervia* (100 mg/kg/day) on healing of ulcers produced by acetic acid injection into the stomachs of rats.

**Treatment (p.o)**	**Dose (mg/kg)**	**Macroscopic analyses (mm^2^)**		**Histological analyses (μm)**	
		**External lesion area**	**Internal lesion area**	**Normal**	**Regeneration area**
**Control**	-	1.10 ± 0.18	0.31 ± 0.07	437.63 ± 31.87	859.98 ± 73.55
**Cimetidine**	100	0.72 ±0.13	0.21 ±0.04	464.06 ± 24.35	858.87 ± 41.58
**EAF**	100	0.62 ± 0.21	0.21 ± 0.03	545.61 ± 22.61 *	740.99 ± 38.12

Results are mean ± S.E.M. n = 6. ANOVA followed by Dunnett's test.

* *p* < 0.05.

**Table 3.** Number of neutrophils and vessels in regenerative mucosal regions in rats with acetic acid-induced ulcer after a 14-day treatment with ethyl acetate fraction (EAF) from *Alchornea triplinervia* (100 mg/kg/day).

**Treatment (p.o.)**	**Dose (mg/kg)**	**Neutrophils (cells/mm^2^)**	**Vessels (vessel/mm^2^)**
Vehicle	-	79.99 ± 21.13	133.24 ± 14.66
Cimetidine	100	142.40 ± 11.59 *	171.31 ± 7.57 *
EAF	100	192.84 ± 18.83 **	219.50 ± 9.39 **

Results are mean ± S.E.M. n = 6. ANOVA followed by Dunnett's test.

* *p* < 0.05,

** *p* < 0.001.
